# Interdisciplinary analysis of drugs: Structural features and clinical data

**DOI:** 10.1017/cts.2022.375

**Published:** 2022-04-29

**Authors:** Daniel Austin, Kumari Biswas, Kailee Pollock, Linh Nguyen

**Affiliations:** School of Pharmacy, Lake Erie College of Osteopathic Medicine, Erie, PA, USA

**Keywords:** Proof-of-concept, team science, interdisciplinary, drugs, chemistry, adverse reactions

## Abstract

**Background:**

Chemical structure is a vital consideration early in the drug development process. Its role in analysis of safety and efficacy is relatively diminished after drugs are approved for clinical use. This interdisciplinary study explores a strategy by which readily available clinical data may be used along with structural features of drugs to identify associations with potential utility for both clinical decision-making and drug development.

**Methods:**

Chemical functional groups and structural groups (SGs) of 261 drugs were manually classified in tiers, and their incidence of gastrointestinal (GI) and central nervous system (CNS) adverse drug reactions (ADRs) were obtained from a clinical database. Drugs with an GI or CNS ADR incidence of at least 10% were analyzed for correlations with their functional and SGs.

**Results:**

Eight statistically significant associations were detected by preliminary analysis: piperazine and methylene groups were associated with higher rate of CNS ADRs; while amides, secondary alcohols, and di-substituted phenyl groups were associated with lower rates of GI or CNS ADRs or both.

**Conclusions:**

Although further study is necessary to understand these associations and build upon this strategy, this exploratory analysis establishes a methodology by which chemical properties of drugs may be used to aid in clinical decision-making when choosing between otherwise equivalent drug therapy options, as the presence of specific groups on drugs may be associated with increased or decreased risks of specific ADRs.

## Introduction

**Fig. 1.**
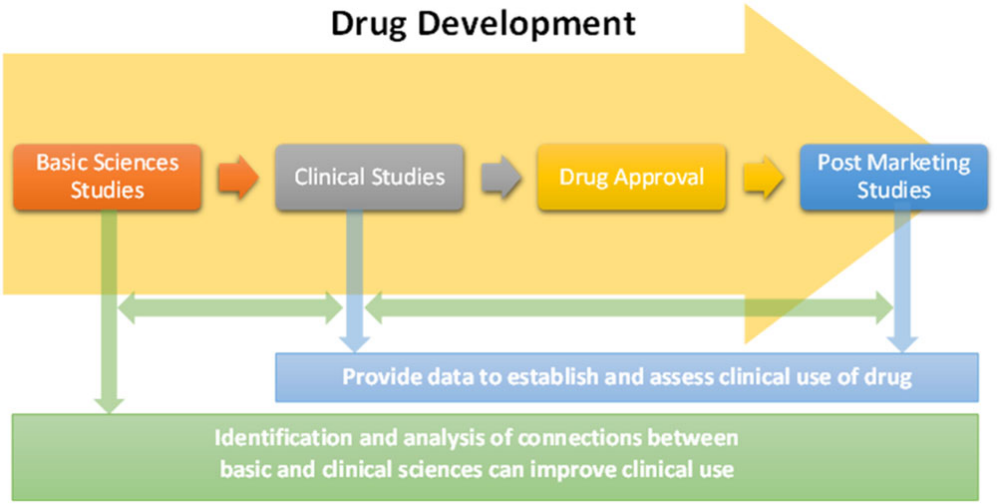
Relationships between basic and clinical sciences in drug development and clinical use.

Drug development and drug therapy require input from multiple basic and clinical scientific disciplines. Basic sciences drive the interdisciplinary drug development process, and clinical performance of a drug is not only the result but also provides outcomes data that is used to optimize the drug development process [1]. The relationship of basic and clinical sciences is therefore “two way” for the drug development process (i.e., basic sciences are used to develop drugs that produce clinical outcomes, and clinical data may be analyzed to improve conceptual understanding of the basic sciences used to develop the drugs). The role of basic sciences in clinical use of drugs is generally limited to prerequisite training for clinical sciences, use of narrow therapeutic index drugs, and retrospective analysis of unexpected outcomes of drug therapy [2–4]. Identification of connections within available scientific and clinical drug data is a means of developing new insights into potential roles of basic sciences in the clinical decision-making process, analogous to their relationship in the drug development process. This interdisciplinary relationship is visualized in Fig. 1. Functional groups (FGs) and adverse drug reactions (ADRs) are ideal variables by which to develop and test this methodology; they are universal, readily available basic and clinical science parameters, respectively, of drugs. Analysis of connections between *in vivo* activity and structural features of drugs has contributed to conceptual understanding in pharmacology. Lipinski’s rule of five is an example of correlations of structural characteristics of drugs to their pharmacokinetics in humans, including absorption, distribution, metabolism, and elimination [5]. FGs impart chemical and physical characteristics of organic and organometallic substances, and they are determinants of pharmacophores, chemical properties of drugs, and drug interfacing with biological systems [6]. Analysis of FGs and features of drugs by Mao et al. identified and evaluated chemical criteria including small FGs, proportion of heavy atoms, and ring systems across drugs of multiple categories. FG associations identified included hydroxy, carboxylic acid, and ester as the most common FGs of drugs, correlation of presence of fluorine and drugs used to treat conditions of the central nervous system (CNS), and correlation of primary amines with antimicrobial and antineoplastic drugs [7]. The International Union of Pure and Applied Chemistry (IUPAC) Gold Book defines FGs as “an atom, or a group of atoms that has similar chemical properties whenever it occurs in different compounds. It defines the characteristic physical and chemical properties of families of organic compounds” [8]. The algorithm developed by Ertl demonstrated broad applicability for identification of organic FGs and offers potential for comprehensive, precise FG analysis of drug molecules [9].

ADRs are important, ubiquitous clinical outcomes with readily available data and rate of incidence. ADRs, also known as side effects (SEs), are unintended and unwanted physiological responses experienced by patients following drug administration [10]. ADRs are a type of adverse drug event (ADE) associated with appropriate medication use. The World Health Organization (WHO) definition of ADR is “a response to a drug which is noxious and unintended, and which occurs at doses normally used in man for prophylaxis, diagnosis, or therapy of disease or for the modification of physiologic function” [11]. ADRs fit into one of three potential categories [12]:

1. Dose-dependent augmentation of pharmacological activity beyond the desired effect
2. Known properties of chemical moieties within drugs associated with specific unwanted chemical or pharmacological effects unrelated to the desired effect
3. Not well understood mechanistically in some or all situations

The role of FGs in causing toxicity to biological systems has been studied by computational, quantitative structure–activity relationship (QSAR), reaction pathways, and structural units and fragments analyses [13,14]. Such analyses are conducted with respect to specific modes toxicity. For example, Wang, et al. identified FGs and clusters of FGs, such as fused aromatic systems, nitrogen heterocycles, and epoxides to be associated with mutagenicity [15]. The US Environmental Protection Agency (EPA) lists FGs and structural fragments associated with various modes of toxicity such as azides and nitroso groups (toxicity caused by electrophiles), phenothiazines (toxicity caused by free radical formation), amines (toxicity to the kidney), ketones (neurotoxicity), carbamates (genetic toxicity), and many others [16]. These noted FGs or clusters of groups all occur in at least one FDA-approved medication, which demonstrates the importance of dose and chemical context to its role in generating toxicity. As this study was designed to detect patterns of toxicity within the context of commonly used medications, the top 300 drug list was selected as an ideal sample of molecules regarding FG exposure (i.e., average dosage ranges) and molecular scaffolding (i.e., biologically privileged structures that met safety standards for FDA approval).

The preclinical drug discovery process aims to produce drug candidates that are both safe and efficacious. Advances in technology, methodology, and available data have contributed to improvements in predicting potential safety issues with drug candidate molecules [17,18]. Despite these advances, failure of drug candidates due to safety-related issues is still a significant challenge [19]. Information obtained from correlative analytical strategies to predict incidence of ADRs has shown potential to aid clinicians and scientists, respectively, in making therapeutic decisions and in identifying agents more likely to succeed in drug development [20]. Despite inability to provide definitive casual information, correlations between basic and clinical sciences such as FGs and ADRs can serve two important functions:

1. Provide additional tools for clinicians for drug therapy selection and evaluation.
2. Provide information for scientists to generate or study pharmacological hypotheses.

ADRs were specified at a system-level, and gastrointestinal (GI) and CNS were selected as optimal systems to establish proof of concept. The digestive and nervous systems were identified for analysis based on high incidence of ADRs (113 and 81 common GI and nervous system/psychiatric disorders listed, respectively, in addition to ADRs shared with other systems) and on potential for enhanced FG discrimination in that these systems possess unique biochemical environments with biological functions associated with managing exposure to xenobiotics (e.g., gastric acid, enzymes, and the blood–brain barrier) [21]. This study therefore presents and utilizes this strategy to explore methodology by which correlations may be identified from existing clinical and structural drug data.

## Materials and Methods

**Fig. 2.**
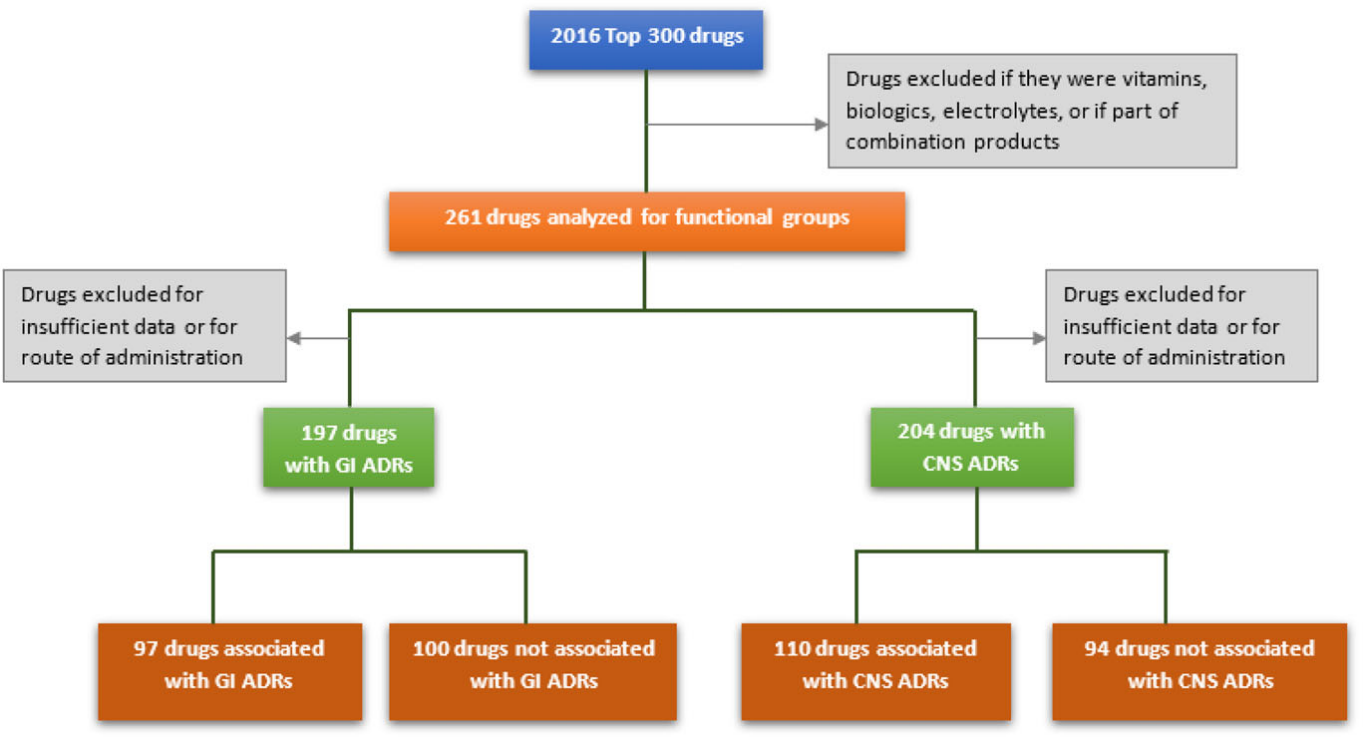
Protocol for preliminary analysis. GI = gastrointestinal; ADRs = adverse drug reactions; CNS = central nervous system.

Medications of the 2016 top 300 drugs list, which comprise approximately 97% of outpatient prescriptions filled in the USA, were evaluated for initial analysis of FGs and ADRs [22,23]. Drugs containing the same active pharmaceutical ingredient (API) with more than one formulation, medications containing combination products already present on the list as single medications, electrolytes, vitamins, and medications classified as biologics or nonsmall molecules were excluded from analysis. These restrictions decreased the sample size to 261 eligible medications. Drugs were classified using the American Hospital Formulary Service (AHFS) Pharmacologic-Therapeutic Classification system into general categories and subcategories for secondary analysis [24]. Follow-up study of significant FG-ADR associations was conducted using medications of the US Veterans Affairs 2018 national formulary list [25]. Drugs were excluded if contained within or met exclusion criteria of initial analysis, resulting in an additional 231 eligible drugs. These drugs were analyzed only for groups significantly associated with ADRs from initial study. The protocol for initial analysis is shown in Fig. 2.

**Fig. 3.**
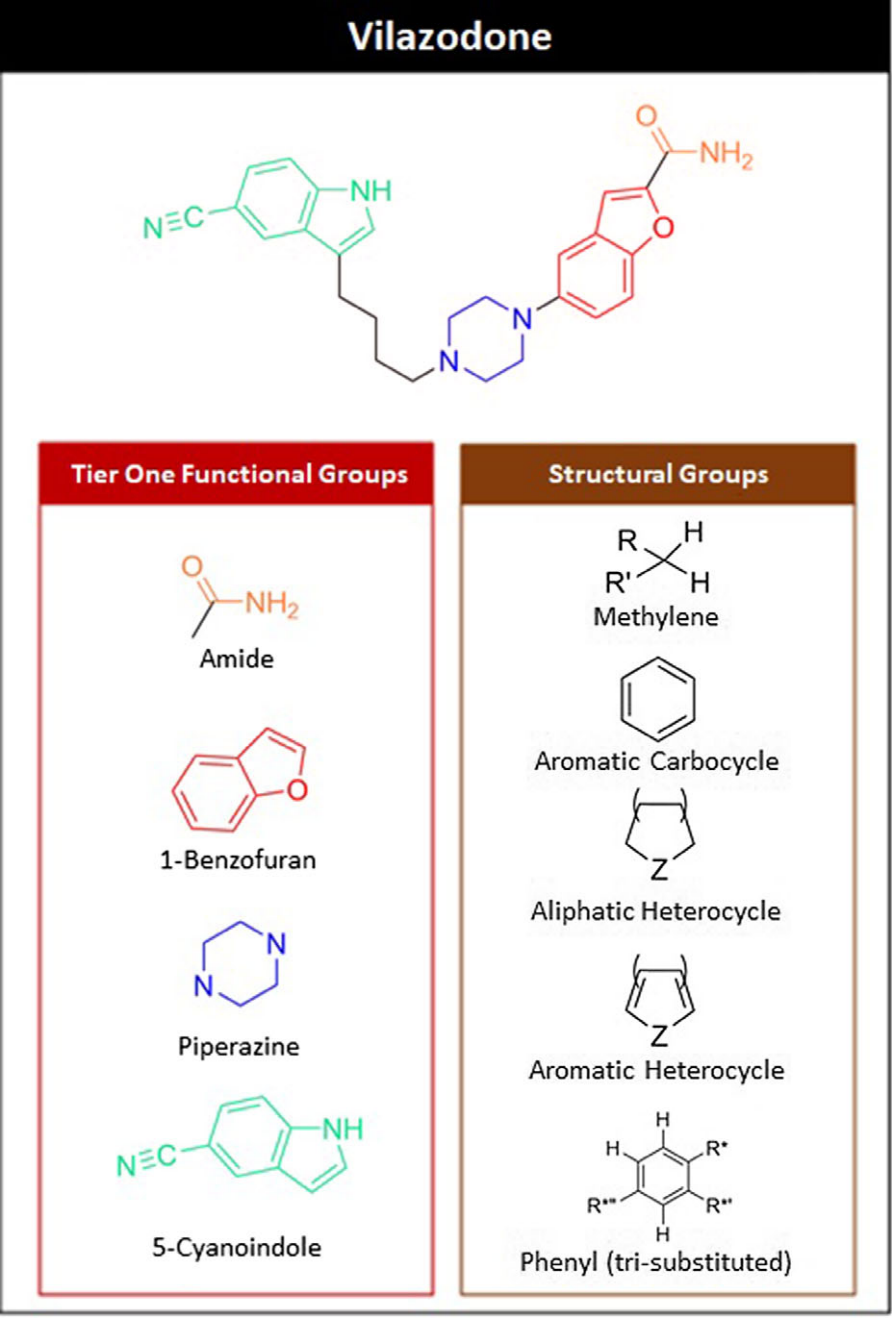
Functional and structural groups of vilazodone. R = carbon substituent; Z = heteroatom.

FGs were identified manually for each medication in “tiers” using an approach derived from the Ertl algorithm [8]. Analysis was conducted on parent molecules only (i.e., without regard to activity of prodrugs). Tier one FGs were nonredundant; each functionalized atom occurred in as few total FGs as possible (usually only one FG, at most two), and all functionalized atoms of the drug molecule were accounted for by the fewest possible total number of FGs. Tier two analysis was comprehensive, and as many FGs as possible were identified. Tier two therefore included all FGs of tier one, as well as comprising groups. Polycyclic groups were labeled as the largest possible cluster of atoms with an existing IUPAC or trivial name, and all comprising groups of ring systems were included in tier two. All nonfunctionalized carbons (i.e., carbons bonded only to carbons or hydrogens), and single-carbocyclic systems were identified as structural groups (SGs). A total of 18 structural criteria were analyzed. Figure 3 demonstrates application of this methodology for the antidepressant drug vilazodone. A comprehensive description of FG and SG analysis methodology and analysis of the initial 261 drugs and FGs is contained in supporting information.

An ADR was defined categorically as associated with a drug if listed as “very common” or greater than 10% incidence by either clinical databases, prescribing information, clinical review articles, or meta-analyses. Drugs were excluded for minimal penetration to systemic circulation (i.e., topical or local delivery) or if quantitative data were not available. In addition to qualitative study, the average daily exposure range of drugs was estimated by calculating moles of drug delivered based on the largest and smallest daily dosing recommendations for FDA-approved indications. All ADRs described as “GI” and “CNS,” except toothache (GI), were included if accordingly specified. Statistical analysis of FG associations with ADRs was conducted by Pearson’s chi-squared test and Fisher’s exact test. Analysis of daily exposure was conducted by independent samples t-tests. All drugs included in analysis, results of FG and ADR analysis, drug classification, and analysis of drug exposure are available in supporting information.

## Results

A total of 258 unique FGs were identified for 261 agents of the top 300 drugs. GI ADRs were associated with 97 of 197 drugs, and CNS ADRs were associated with 110 of 204 drugs. The most common categories of the top 300 medications were CNS agents, cardiovascular drugs, anti-infective agents, autonomic drugs, and hormones and synthetic substitutes.

**Fig. 4.**
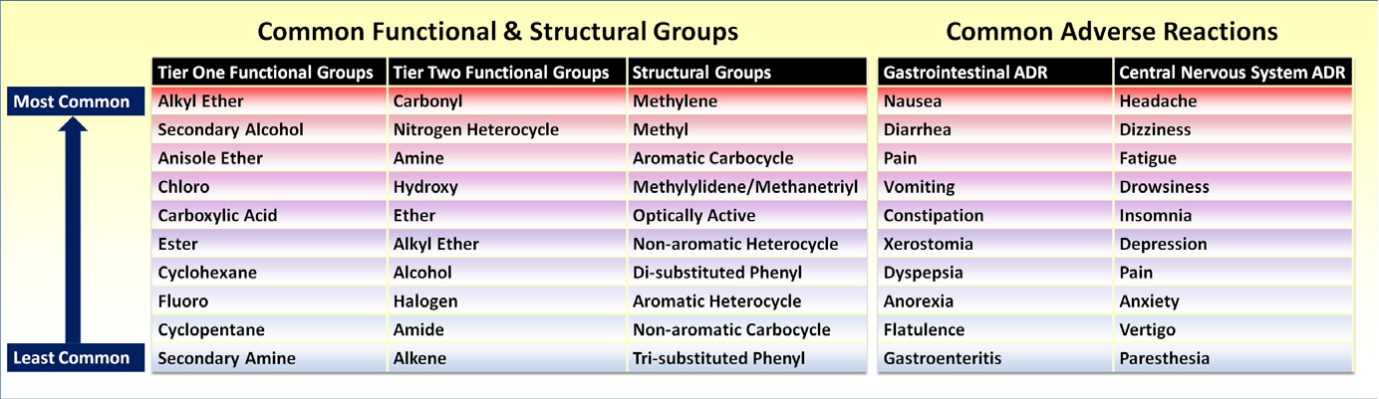
Most common functional groups and ADRs of the 2016 top 300 drugs. ADRs = adverse drug reactions.

**Fig. 5.**
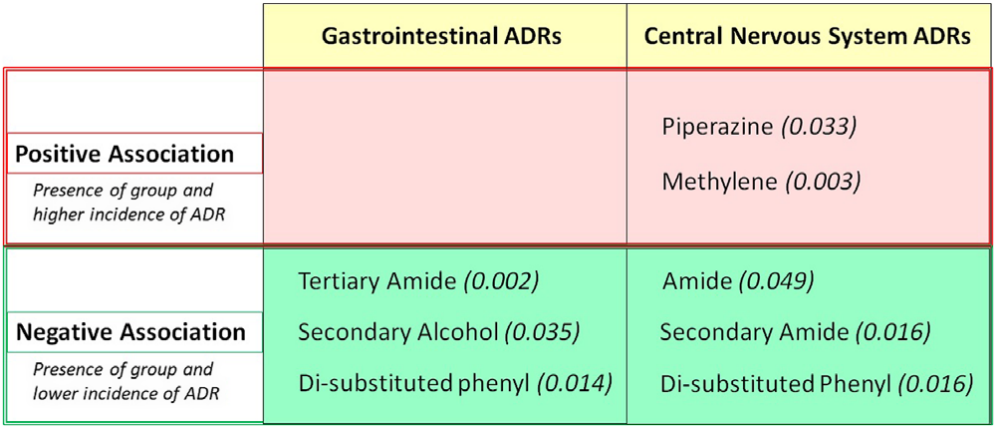
Significant associations identified in preliminary analysis (*p*-values). ADRs = adverse drug reactions.

The most common FGs and ADRs are shown in Fig. 4. Comprehensive results of analysis are available in supporting information. All tier one FGs, tier two FGs, SGs, and estimated ranges daily exposure to drug were analyzed for associations with GI or CNS ADRs. Associations were defined as positive if presence of a group correlated with occurrence of ADR and were defined as negative if presence of a group correlated with absence of ADR. Initial analysis detected eight significant associations: three groups for GI ADRs and five groups for CNS ADRs (*p* < 0.05). Significantly associated groups are contained in Fig. 5. Of 231 additional medications, 164 were analyzed for the groups that demonstrated significant associations. GI and CNS ADRs were associated with 104 and 88 drugs, respectively. Significant correlations were maintained for three groups and one group for CNS and GI ADRs, respectively. Results of follow-up analysis are contained in Table 1.

**Table 1.** Associations determined by follow-up analysis

ADR category	Functional or structural group	Pearson’s correlation	*p*-Value
**CNS (*n* = 368)**	Amide	−0.152	**0.003**
	Secondary Amide	−0.147	**0.005**
	Tertiary Amide	−0.108	**0.038**
	Piperazine	0.094	0.073
	Secondary Alcohol	−0.120	**0.022**
	Di-substituted Phenyl	−0.043	0.407
	Methylene	0.106	**0.042**
**GI (*n* = 361)**	Amide	−0.035	0.513
	Secondary Amide	0.052	0.320
	Tertiary Amide	−0.096	0.070
	Piperazine	−0.001	0.989
	Secondary Alcohol	−0.086	0.104
	Di-substituted Phenyl	−0.131	**0.013**
	Methylene	0.011	0.834

ADR = adverse drug reaction; CNS = central nervous system; GI = gastrointestinal.

Significance level < 0.05.

## Discussion

Concurrent chemical and clinical analysis of drugs provides an additional means by which clinicians may use chemical structure in clinical decision-making and may contribute to understanding of effects of comprising structural features *in vivo*. The effect size of this mode of analysis was small (e.g., the largest association detected in this study was amide and CNS ADRs, −0.152). A possible explanation for this observation is that the intrinsic role of a given FG in causing an ADR is likely to be secondary to other factors such as specific binding interactions of pharmacophores. As drug molecules are irreducible combinations of FGs, the effects of a given group may therefore be masked, augmented, or otherwise confounded by the other groups of the drug molecule. An alternative explanation is that the impacts of single FGs are nonsignificant relative to other factors to the extent that associations detected are largely random. To address the latter explanation, a large quantity of drugs and clinical data obtained from large studies and multiple sources were utilized to maximize sample size and minimize the probability of random error. Results suggest that CNS ADR associations, which were largely maintained through follow-up analysis, may be attributed to the former explanation, while the majority of groups initially associated with GI ADRs did not demonstrate significance in follow-up analysis and are more likely to be inconsequential results.

Relationships between structure and clinical effects yet undiscovered are therefore likely to be subtle and context-dependent, and the chemical environment experienced by drugs and clinical effects they produce are complex, multifaceted, and composite outcomes with substantial potential for confounding. This mode of analysis, with its large sample size and comprehensive, hierarchical chemical group analysis, is designed to detect such relationships. For example, the detection of effects due to an unknown pharmacophore, masked by the effects of another frequently occurring pharmacophore (e.g., the former being structurally nested within the latter), could be detected through this methodology. Variance within clinical data due to inherent subjectivity and imprecision may be larger than effect size and increases probability of false-positive results. Therefore, the clinical parameter (ADRs in this case) must be set at a high threshold. This study defined an ADR as 10% or greater to optimize the “size of the net” in detecting associations. This high threshold decreases probability of detecting associations overall but increases the probability of associations being nonrandom and clinically relevant if detected.

A major determinant of the generalizability of this methodology is availability of data, and most of the ADR information was obtained from a clinical database (Lexicomp®) [26]. Clinical databases feature readily accessible data that is derived from pooling of multiple sources and studies, and the large sample sizes decrease confounding [27]. The extensive usage and prior clinical study of the top 300 drugs maximized availability and generalizability of clinical data. Common, system defined ADRs of preferably idiopathic nature and of general clinical significance were desired for analysis of FG correlation to increase the probability of identifying useful associations. To conduct comprehensive and reproducible chemical analysis, FGs were identified in tiers, and SGs were included in analysis. Tier one groups were less prevalent and less redundant; each functionalized atom was contained in as few FGs as possible (usually only one, at most two), and tier two included all FGs of tier one, as well as comprising groups.

A notable discovery of this work is negative correlation of amides with CNS ADRs. The EPA lists chemical groups, classes, and elements as potential neurotoxins [16]. Several groups listed, such as ketones and nitriles, are present on drug molecules and were not significantly associated with CNS ADRs. Additionally, aryl- and acrylamides are listed, and amides in general were negatively correlated with CNS ADRs in this study. These findings demonstrate the complexity of the role of chemical structure, and the importance of chemical context and dose, in determining toxicity. Although further study is required to corroborate and explain these results, exploration of application to medication therapy management demonstrates the utility of this methodology. A clinician selecting, for example, between two otherwise equivalent medications (i.e., both first-line options recommended by pertinent guidelines, similar cost and availability, identical dosing schedule, similar ADR profile, etc.) could use the structures of the drugs themselves along with this information to identify a patient-specific therapy based on the presence or absence of FGs or SGs associated with the presence or absence of ADRs. For example, the atypical antipsychotics ziprasidone and quetiapine have similar ADR profiles, including multiple CNS ADRs. Ziprasidone features a secondary amide, which suggests lower overall risk of CNS ADRs and is corroborated by listed frequency of incidence less than 10%.

### Limitations and Future Research

There are limitations to this mode of analysis. Fundamental issues with this FG and SG analysis include dichotomous listing (i.e., present or not present), which may not account for potential effects due to multiple occurrences of a FG on the same drug molecule. A limitation of construct is that pharmacophores comprised of clusters of FGs are the smallest chemical units associated with many biological effects produced by molecules, and their biological activities in such situations cannot be deconstructed (i.e., all comprising FGs must occur concurrently for effects associated with specific pharmacophores). Additionally, rare FGs are reasonably expected to occur in clusters of pharmacophores and toxicophores within medication classes. These limitations were factored into the rationale of the design of this study, in that FGs themselves may be viewed as the simplest possible pharmacophores, and analysis is necessarily hierarchical (e.g., an unknown pharmacophore may be contained within the structure of a larger pharmacophore). A significant limitation of this study is attrition due to insufficient data or exclusion. Most medications lacking quantitative data are agents that were approved prior to implementation of modern protocols, with the availability of clinical information therefore relegated to reviews, case reports, and results of their uses as comparator drugs. This limitation may be addressed by identifying additional modern agents possessing relevant clinical data to increase the initial pool of drugs for analysis. This study, which relies upon many samples with normal distribution of pharmacological parameters, intentionally does not account for pharmacodynamics, pharmacokinetics, or any effects due to sterics or clustering patterns of multiple FGs as the effects of individual FGs are anticipated to be smaller than these covariates. These challenges and limitations present significant potential for confounding, which may be addressed by future analysis through the use of technology, prudent study design, increased specificity of outcomes, and increased sample size.

## Conclusion

The role of basic sciences is vital during the drug development process but diminishes as the drug moves through clinical study and post-marketing analysis. Once approved for clinical use, associations between physicochemical properties and clinical effects may not be considered by clinicians. This study explores methodology by which basic sciences may be applied to clinical situations (i.e., specific structural and FGs within drug molecules correlated with adverse reactions (ADRs) of drugs) and provides the basis for further research to determine how to best obtain and use this information for both drug development and drug use in practice. Key considerations for further development of this methodology include determination of clinical parameters to study, how to use associations to develop or build upon paradigms in pharmacology and medicinal chemistry, and how to use this information to optimize drug therapy outcomes for patients.
